# Resistant energy analysis of self-pulling process during dropwise condensation on superhydrophobic surfaces†

**DOI:** 10.1039/c8na00237a

**Published:** 2018-12-20

**Authors:** Aref Vandadi, Lei Zhao, Jiangtao Cheng

**Affiliations:** Department of Mechanical and Energy Engineering, University of North Texas Denton TX 76207 USA.; Department of Mechanical Engineering, Virginia Polytechnic Institute and State University Blacksburg VA 24061 USA chengjt@vt.edu

## Abstract

Recently the development of superhydrophobic surfaces with one-tier or hierarchical textures has drawn increasing attention because enhanced condensation heat transfer has been observed on such biomimetic surfaces in well-tailored supersaturation or subcooling conditions. However, the physical mechanisms underlying condensation enhancement are still less understood. Here we report an energy-based analysis on the formation and growth of condensate droplets on two-tier superhydrophobic surfaces, which are fabricated by decorating carbon nanotubes (CNTs) onto microscale fluorinated pillars. Thus-formed hierarchical surfaces with two tier micro/nanoscale roughness are proved to be superior to smooth surfaces in the spatial control of condensate droplets. In particular, we focus on the self-pulling process of condensates in the partially wetting morphology (PW) from surface cavities due to intrinsic Laplace pressure gradient. In this analysis, the self-pulling process of condensate tails is resisted by adhesion energy, viscous dissipation, contact line dissipation and line tension in a combined manner. This process can be facilitated by adjusting the configuration and length scale of the first-tier texture. The optimum design can not only lower the total resistant energy but also favor the out-of-plane motion of condensate droplets anchored in the first-tier cavity. It is also shown that engineered surface with hierarchical roughness is beneficial to remarkably mitigating contact line dissipation from the perspective of molecular kinetic theory (MKT). Our study suggests that scaling down surface roughness to submicron scale can facilitate the self-propelled removal of condensate droplets.

## Abstract 2

Resistant energy analysis of self-pulling process during dropwise condensation on superhydrophobic surfaces with optimum surface roughness.

## Graphical abstract

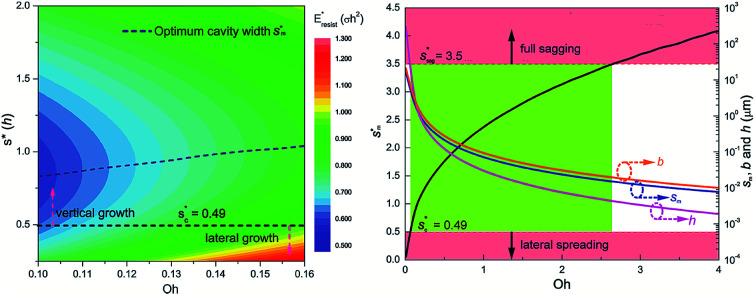

## 1. Introduction

During dropwise condensation on a superhydrophobic surface with micro/nano-structures, condensate droplets can form and grow in three types of morphology, *i.e.*, the Cassie state,^1^ the Wenzel state,^2^ and the partially wetting morphology (PW).^3^ A Cassie-state droplet stands on top of surface roughness and is characterized by a large contact angle, small contact angle hysteresis (<5°) and exceptional mobility. These features could lead to self-propelled removal of condensate droplets and consequently facilitate rapid purging of the condenser surface. Nonetheless, the air cushion underneath a Cassie droplet impedes efficient heat transfer. By contrast, a droplet in the Wenzel state^4,5^ impenetrates surface cavities to facilitate heat transfer between the condensates and the condenser surface, but exhibits strong adhesion to the structured surface. Different from the Cassie state and the Wenzel state, droplets in the PW morphology only partially fill the cavities underneath^3^ or just the first-tier cavities in hierarchical roughness, *i.e.*, micro-Wenzel nano-Cassie state. In this case, the PW condensates can form a relatively efficient thermal conduction path *via* bridging the main gap between the condensates and condenser surface, and in the meanwhile remain comparatively mobile to avoid condensate flooding on the condenser surface. Zhang *et al.* studied dropwise condensation on nanostructured microporous surfaces in environmental scanning electron microscope (ESEM) and observed the self-pulling of a single PW droplet without coalescence and coalescence-induced PW to Cassie state transition, *i.e.*, PW-Cassie transition.^6^ Therefore, the PW morphology for condensate droplets is desired for continuous dropwise condensation (CDC) with enhanced heat transfer.^7,8^

Achieving sustained dropwise condensation on micro/nano-engineered surfaces places stringent requirements on the proper design of the configuration and length scale of surface structures and necessitates meticulous control of nucleation density and droplet morphology, which entail deep understanding of the underlying mechanisms governing condensate growth dynamics. Recently droplet jumping condensation on a nanostructured superhydrophobic CuO surface was demonstrated to enhance heat transfer efficiency by 30%.^8^ However, these condensation experiments were conducted in carefully-tailored and mild thermal conditions with a low supersaturation (<1.12) and a relatively low critical heat flux (<8 W cm^−2^). Otherwise, condensate flooding on the condenser surface would occur and give rise to the unacceptable rise of thermal resistance thereon. Based on interfacial energy analysis, Rykaczewski *et al.* reported that the growth mechanism of individual condensate microdroplets on nanotextured surfaces is universal and independent of the surface architecture.^9^ The key role of the nanoscale topography is to confine the base area (*i.e.*, wet spot) of forming embryos, which allows droplets to grow only through contact angle increase. Through observing condensation on one tier of nanowires in ESEM, they claimed that the formation of condensate nuclei could be controlled close to the top of the nanostructures.^9^ But how to form discrete condensate distribution on the surface of nanowires postponing flooding as well as validation of condensation heat transfer enhancement were not systematically studied in this work. Up to date, the rationale for designing biomimetic surfaces with optimum configuration of surface roughness in real thermal conditions are still lacking. Most of such reported condenser surfaces^3–11^ are characterized by fortuitous design and configuration, rather than driven by the fundamental principles of thermal and physical processes. In order to achieve sustained dropwise condensation on an engineered surface, it is imperative to understand the formation of condensate embryos, nucleation site and condensate density distribution, the dynamic growth of condensate droplets and evolution of droplet morphology.^3^

In this paper, we report our energy-based analysis of growth dynamics of dropwise condensates on biomimetic surfaces with two-tier hierarchical textures and their structural optimization. In particular, we focus on the intrinsic energetics associated with the transition of a condensate tail, which is entrapped in the first tier cavity but exhibits Cassie state relative to the second-tier nanoscale roughness (exclusively referred to as the PW condensates in this work), to the Cassie state through the self-pulling mode without coalescence with neighbouring droplets. In this analysis, the resistant energy associated with the expulsion of a condensate tail in the PW morphology is divided into three main parts. The first part is the adhesion energy due to the adhesion of the tail to the nanotextures in the first tier cavity. The second part is the viscous dissipation during the upflow of the condensate tail from the first tier cavity to the bulky portion of the PW droplet. The third part is related to contact line dissipation that occurs within three phase (liquid/vapor/solid) contact zone of the condensate tail. Besides, the effect of line tension on condensate state transition is also discussed. By minimizing the energetic resistance, the first tier roughness is optimized to favour the PW-Cassie transition of a condensate tail in the cavity. This work can advance our understanding on the growth dynamics of PW condensate droplets and aid the design of micro/nano-engineered lotus-leaf-like condenser surfaces promoting continuous dropwise condensation.

## 2. Experimental methods

**Fig. 1:**
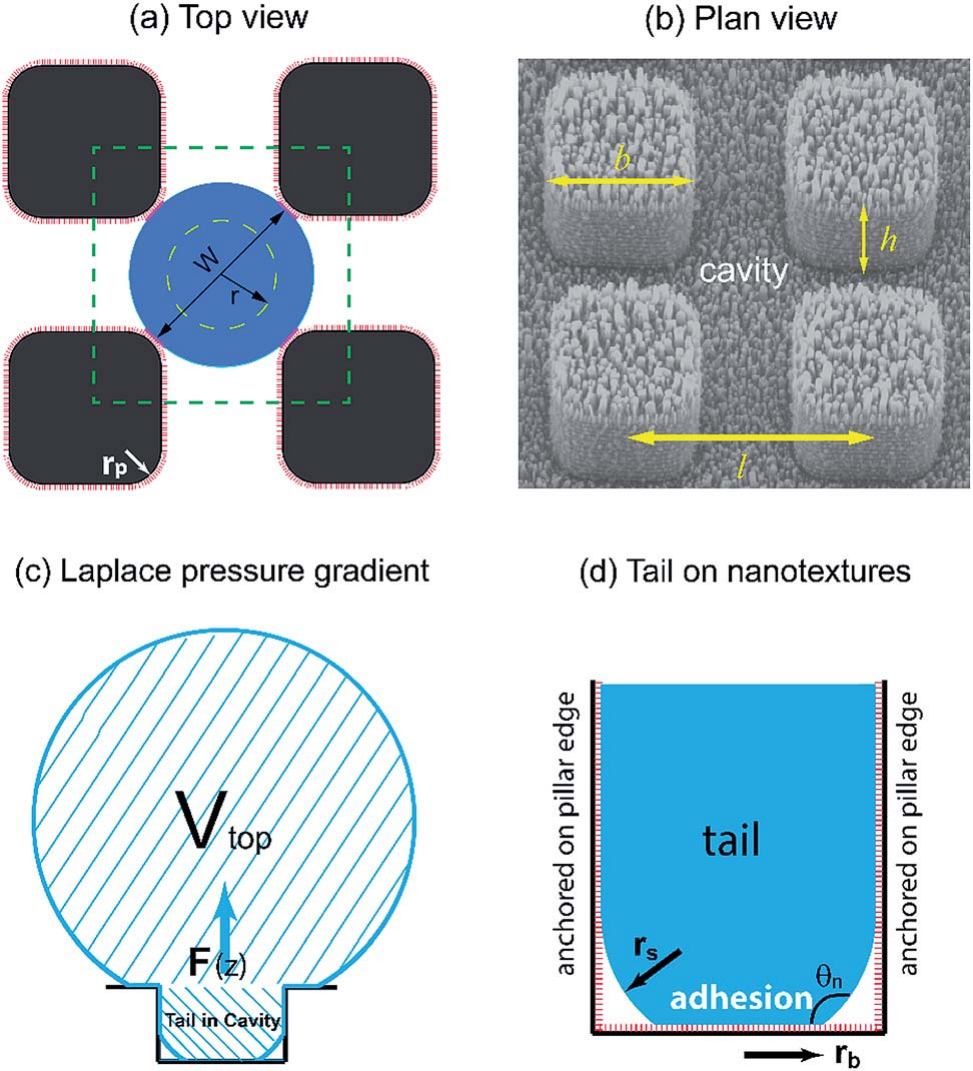
(a) Schematic (top view) of the first tier pillar array on a two-tier textured surface with the second tier nanotextures shown in red bars. The four short pink segments indicate that the condensate droplet formed in the cavity anchors on the vertical corner edges of the four surrounding pillars. (b) SEM image (plan view) of a cavity cell confined by the surrounding first tier pillars, which are decorated by CNTs. (c) Side view of a condensate droplet in the PW morphology. The tail can enhance heat conduction by bridging the gap between the condensate bulk and the cavity base. For simplicity, only one tail out of multiple tails is shown under the droplet. In the self-pulling mode, out-of-plane expulsion of the condensate tail is intrinsically driven by Laplace pressure gradient. (d) The base of the condensate tail is adhered to the nanotextures in the cavity valley (surface contact) while the tail body is in contact with the nanotextures along the first tier vertical edges. Droplet portion above pillars is not shown for the purpose of simplicity. It is assumed that nucleation embryos start growing close to the top portion of the nano-structures forming a wet spot and therefore as-grown condensate microdroplet is at the Cassie state relative to the nanoscale roughness.

In our dropwise condensation studies we fabricated biomimetic lotus-leaf-like surfaces with two-tier hierarchical structures as shown in Fig. 1(a) and (b).^12^ Regularly-positioned silicon micropillars^12–16^ were adopted as the first-tier texture, *i.e.*, the primary roughness, and carbon nanotubes (CNTs) were decorated on the micropillars as the second-tier texture, *i.e.*, the secondary roughness. The width, height and center-to-center pitch (period) of the first tier pillars are denoted by *b*, *h* and *l*, respectively, while *r*_p_ stands for the corner radius of the first tier pillars. In this work, the first tier has a solid fraction of *f*_f_ = *b*^2^/*l*^2^ ≈ 0.32 and the second tier nanostructures has a solid fraction of *f*_n_ ≈ 0.25, so the solid fraction of the two-tier textures is *f* = *f*_f_*f*_n_ = 0.08, which is much smaller than 1.^11,12^ As-formed micro/nanostructured surface was conformally coated by a thin layer of fluoropolymer (FluoroPel™ PFC1601V, Cytonix or Teflon®) to strengthen its hydrophobicity to superhydrophobicity. This type of engineered surfaces with regular geometries provides an ideal platform to study the dynamics of droplet growth and expulsion of the tail of a PW condensate droplet in a unit cell, *i.e.*, the cavity volume surrounded by four micropillars as shown in Fig. 1(b), as well as the spatial distribution of condensate droplets.

**Fig. 2:**
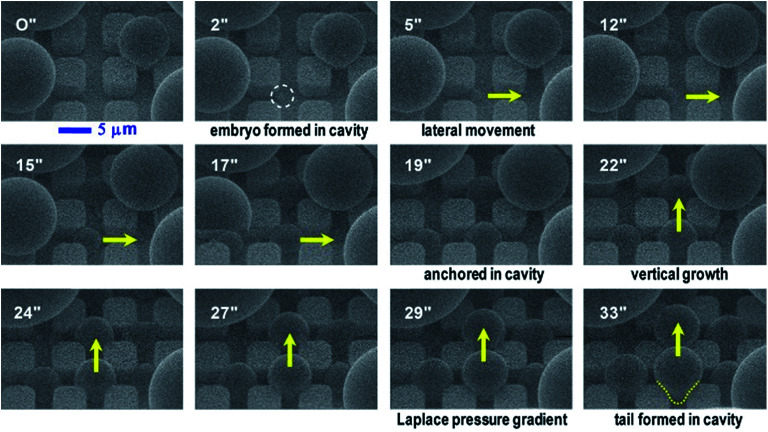
ESEM snapshots (in seconds) of dynamic growth of condensate droplets in the first tier pillar cavities (*b* ≈ 5 μm, *h* ≈ 6 μm, *l* ≈ 9 μm) on the hierarchical superhydrophobic surface. The video was captured at 12.5 kV and a beam current of 0.2 nA. Vapor pressure was ∼5 torr in ESEM. Dotted line in the 33′′ snapshot indicates the tail of a condensate droplet formed in the PW morphology within four neighboring pillars. Hierarchical roughness can effectively control and confine spatial distribution of emerging condensate droplets out of first tier cavities.

Our study of surface roughness optimization starts with the two-tier hierarchical structures as introduced above. Fig. 2 shows a close inspection of condensation process in ESEM. The formation of nucleation embryos in nanostructure cavities and in the first tier pillar cavities is ubiquitous since the critical nucleation radius of water is around tens of nm. The free energy barrier to condensate formation in the roughness cavity, *i.e.*, the change in the free energy associated with embryo formation, could be calculated as:^17,18^1
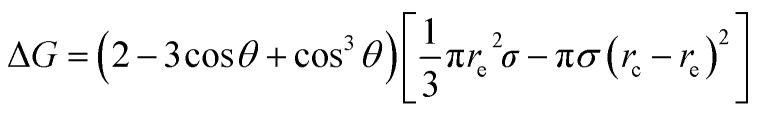
where *r*_e_ = 2*ν*_l_*σT*_w_/*h*_lv_(*T*_sat_(*P*_v_) − *T*_w_) is the equilibrium radius and *σ*, *r*_c_, *θ*, *ν*_l_, *h*_lv_, *T*_w_, *T*_sat_, *P*_v_ are the liquid–vapor surface tension, the characteristic size of formed embryos, the Young's contact angle, liquid specific volume, latent heat, wall temperature, saturation temperature, and vapor pressure, respectively. Assuming *θ* ≈ 115° on fluoropolymer-coated surface, *h*_lv_ ≈ 2500 kJ kg^−1^, *T*_sat_ = 278 K, *T*_w_ = 273.15 K, *σ* ≈ 0.075 N m^−1^, and *ν*_l_ ≈ 0.001 m^3^ kg^−1^, the equilibrium radius *r*_e_ is ∼3 nm in our ESEM experiment. The free energy barrier to form condensate nanodroplets within the CNT interstices of ∼100 nm is about −6.8 × 10^−15^ J. By analogy, the free energy barrier to form condensate microdroplets in the first tier cavities of 4–5 μm wide is about −1.15 × 10^−11^ J. Therefore, it is energetically favourable to form condensate droplets in the first tier cavities while being fed by nucleation embryos formed in the interstices of CNTs. As such, condensate droplets firstly emerged at the bottom edges in the first tier cavities as indicated by the white dotted circle in the 2′′ time frame of Fig. 2 in order to minimize the area of liquid–vapor interface.^4^ The formation of the primitive microdroplets, which seem to root in and sprout from the first tier cavities in spite of ubiquitousness of nucleation embryos on nano-roughness, has been confirmed both in ESEM^12^ and under optical microscope.^11^ Subsequently morphing-induced Laplace pressure gradient worked as the driving force to laterally (in plane) propel the condensate body among the pillars (frame 5′′ to frame 17′′), during which the continuously growing condensates eventually took the shape of sphere (droplet) to maintain a relatively lower surface energy.^4^ During this growing process, the forming condensate microdroplets in the first tier cavities swept and swallowed the surrounding nucleation embryos. Droplet in-plane movement continued until it reached the cavity centre and got anchored by the surrounding pillars (frame 19′′). Then it started to grow upward in the out-of-plane direction (*z* direction) with constrained in-plane lateral spreading (frames 22′′ to 29′′). Eventually it reached the pillar top and evolved over them. Thereby a stretched condensate droplet formed in the PW state with its tail entrapped in the cavity (frame 33′′). Such PW droplets can potentially give rise to enhanced heat transfer^3^ but still need to be expulsed to the Cassie state for agile removal.

**Fig. 3:**
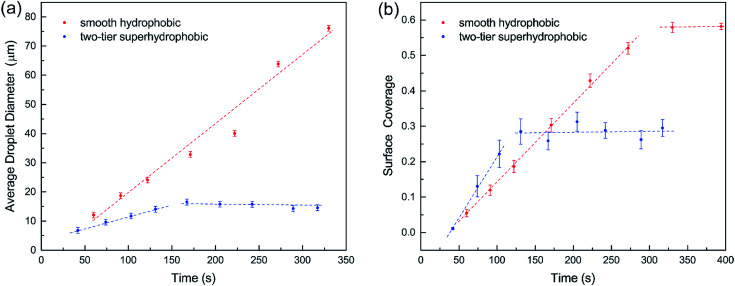
(a) Average droplet diameter and (b) surface coverage in the condensation process on smooth and two-tier surfaces.

Furthermore, we compared condensation process of water vapor on the two-tier superhydrophobic surface in ESEM^12^ with that on a smooth hydrophobic surface, which was formed by spin-coating a thin layer of fluoropolymer (FluoroPel™ PFC1601V, Cytonix or Teflon®) on a silicon wafer. The average sizes of condensate droplets during condensation on the two surfaces were calculated and plotted in Fig. 3(a). On the smooth surface, condensate droplets continuously coalesced with neighbouring condensates forming larger droplets, but the majority of the condensates still remained on the surface (see ESI†). Consequently, the average droplet diameter continuously grew as shown in Fig. 3(a). On the contrary, the average drop diameter on the two-tier surface started growing at the early stage of condensation but reached a saturated value of ∼15 μm during the subsequent stage. This could be explained by the self-cleaning ability of the two-tier superhydrophobic surfaces, which removes the condensate droplets by coalescence-induced jumping.^12^ The surface coverages of condensates on both the smooth and two-tier surfaces are plotted *versus* time in Fig. 3(b). Initially surface coverages on both surfaces increased along with condensation and got flattened out respectively following the early stage. For smooth hydrophobic surface the surface coverage levelled out at 0.58 while the superhydrophobic surface coverage got stabilized at around 0.28, which is almost half of the smooth surface coverage. Maintaining a higher percentage of unwetted bare surface could lead to less thermal resistance towards condensation, which can potentially enhance the rate of condensation and subsequently increase heat transfer on the two-tier structured surface.

## 3. Results

### 3.1 Theoretical models

Regarding a forming PW condensate, its tail is entrapped in the cavity of primary roughness but could be expelled either by self-pulling^6^ because of morphing-induced Laplace pressure gradient (intrinsic mechanism), or jump off *via* coalescence with neighbouring droplets (extrinsic mechanism). Nevertheless, immobile coalescence^13^ has been observed in the event that the released surface energy in coalescence is smaller compared to adhesion work, viscous dissipation consumption and contact line dissipation/pinning.^19^ For the purpose of structural optimization of surface roughness, we are not concerned with interactions (*e.g.*, coalescence) between condensate droplets already sitting on top of surface roughness. Alternatively, we focus on the resistant energy preventing a single condensate tail from transitioning to the Cassie state, which would be the favourable state for agile condensate removal. In our analysis, we divide the total resistance energy into three major parts, *i.e.*, adhesion energy, viscous dissipation and contact line dissipation.

Adhesion energy is one of the main barriers impeding the PW-Cassie transition as discussed in previous studies.^20^ The crucial step to form a mobile Cassie droplet is to first detach the condensate tail from the cavity and subsequently to expel the tail to the top of the pillars (PW-Cassie transition). The surface free energy change during the detachment of the tail per unit surface area would be *W*_adh_ = *σ*(1 + cos *θ*),^21^ where *θ* ≈ 115° is the Young's contact angle on the fluoropolymer-coated (FluoroPel™ PFC1601V, Cytonix or Teflon®)^12^ smooth surface. The contact area of the tail with the nanostructures on the cavity base is *f*_n_π*r*_b_^2^, where 
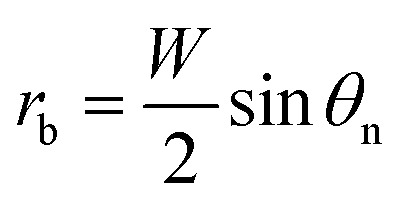
 is the radius of the tail base, *W* = 2(*l* − *b*), and *θ*_n_ ≈ 150° is the contact angle on the second tier nanostructures (Fig. 1(d)). The contact area of the tail along the pillar sidewalls is ∼*L*_cl_*h*, where *L*_cl_ is the length of the three-phase contact line as illustrated by the pink segments in Fig. 1(a) and the derivation of *L*_cl_ can be found in the ESI.† Then the work required to detach the condensate tail from the cavity is

2 *E*_adh_ = *W*_adh_ × *A*_adh_ = *σ*(1 + cos *θ*)*f*_n_(π*r*_b_^2^ + *L*_cl_*h*)

Consequently, actual contact area *A*_adh_ is scaled down by a factor of *f*_n_ due to the secondary nano-roughness.

The viscous dissipation incurred by the internal convection of a PW droplet makes another part of the resistant energy. As evidenced by the nonuniform heat transfer on the base of a condensate droplet, condensate droplets are usually not in a thermodynamically equilibrium state.^22^ The gradually expanding upper portion of a PW condensate leads to a decreasing capillary pressure therein whereas its tail portion remains under high capillary pressure. Thus-generated internal pressure gradient^23,24^ would pull the condensate tail upward, and at a specific size the droplet base would detach from the first tier valley, leading to self-pulling (or self-jumping) of the stretched PW droplet without coalescence. The self-pulling mode has been recently reported by Aili *et al.*^6^ in their study of condensation on nanostructured microporous surfaces. To analyze self-pulling of a condensate tail in a first tier cavity, we assumed Laplace pressure force *F* as the intrinsic mechanism in this study and the magnitude of viscous dissipation during the tail elevation is estimated as^25^3
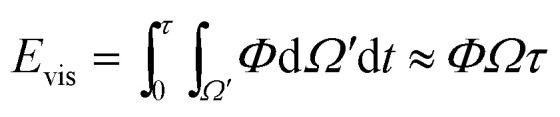
where *Ω* is the volume of the condensate tail and *τ* is the period of viscous dissipation (in the event of coalescence, *τ* can be characterized as the capillary time scale^19^). In the cylindrical coordinate system (Fig. 1(c)), the dissipation function *Φ* with the main flow in *z* direction (out of plane) is approximately given by4
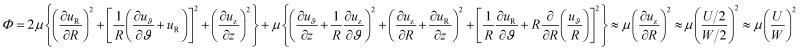
where *μ* is the viscosity of the condensate (water), *R* the radial coordinate (in plane), *ϑ* the azimuthal coordinate, *u*_z_ the out-of-plane velocity, and *U* the eventual vertical velocity of the expulsion process.

Regarding the dynamic growing process of a droplet on the pillar-arrayed surface, the surface tension *σ*, the viscosity *μ* and the inertia govern the liquid motions. For liquid, the ratio of viscous dissipation to surface tension and inertia is characterized by the Ohnesorge number Oh = *μ*/*ρσL*, where *ρ* and *L* are the fluid density and the characteristic length scale (*L* = pillar height *h* in this work, *i.e.*, the height of the condensate tail in the cavity), respectively. In typical vapor condensation experiments, Oh ∼0.1 (*ρ* = 998 kg m^−3^, *μ* = 0.001 Pa s, *σ* ≈ 0.075 N m^−1^ for water at 5 °C and 1 bar, and *L* is on the order of μm) indicating viscous dissipation starts to dominate the surface energy effect^19^ and the self-pulling elevation of the tail is a capillary-inertial process (Oh < 1) in the cavity. Thus the average velocity could be assumed to be *U*/2 and the upward displacement of the mass center of the tail is *h*/2, then the time scale for the PW-Cassie transition can be estimated as 
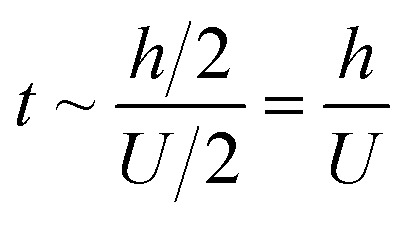
, which is actually scaled as the capillary time (see ESI† for detailed derivation). By the momentum law the velocity of the droplet tail could be scaled as 
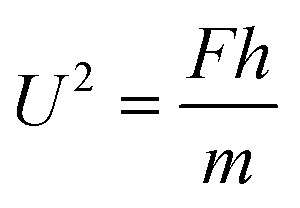
.

Since the size of the entrapped tail is much smaller than the capillary length of 2.7 mm of water, the bottom portion of the tail is assumed to be a spherical cap as shown in Fig. 1(d). So the curvature of the bottom tail can be estimated as *r*_s_ = *W*/2 cos(π − *θ*_n_).^21^ Given the larger radius of the condensate droplet on top (Fig. 1(c)), the pressure in the top portion of the tail should be much smaller than the bottom pressure. Consequently the pressure difference in the tail can be estimated as 
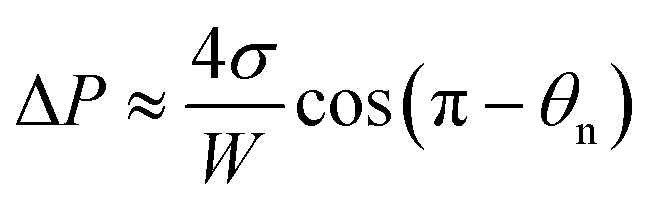
 and the vertical driving force *F* is scaled as5
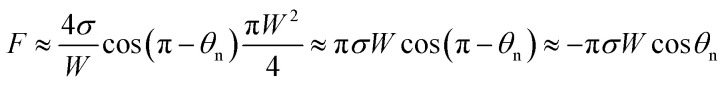


As such, depinning of the solid–liquid–vapor contact line in the cavity occurs when a certain value of critical pressure inside a droplet is surmounted.^26^ With the tail mass *m* ≈ *ρ*π*W*^2^*h*/4, we have 
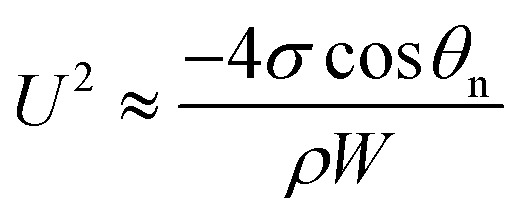
 and the energy loss due to viscous dissipation in the self-pulling process could be estimated as6
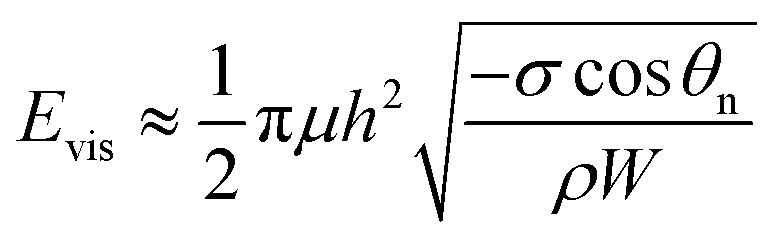


Our vapor condensation study in ESEM^12^ and other moisture condensation studies^11^ have observed extensive immobile coalescence on superhydrophobic surfaces, which can be partially ascribed to contact line pinning and hence contact line dissipation.^27,28^ Therefore, contact line dissipation should be given a careful evaluation in the energy analysis. From the point of view of molecular kinetic theory (MKT),^29–33^ the displacement of the three-phase contact line is determined by the forward and backward jumping frequencies, *K*^+^ and *K*^−^, of liquid molecules in the contact zone. At equilibrium, *K*^+^ = *K*^−^ = *K*_0_, where 
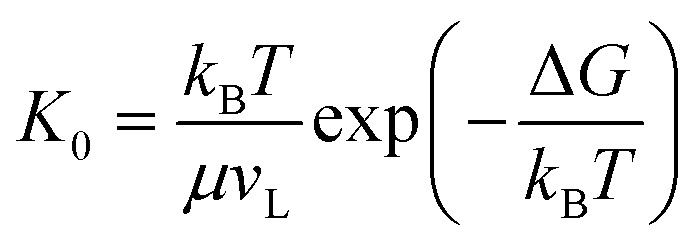
 is the equilibrium displacement frequency, *k*_B_ is the Boltzmann constant, *T* is the absolute temperature, and *v*_L_ is the unit volume of flow of the liquid at the contact line. The activation free energy Δ*G* arises from the solid–liquid interaction, which can be taken as the work of adhesion, and the liquid–liquid viscous effect.^33^ Disturbed by an external driving work (shear stress, capillary force, hydration force, van der Waals interactions, *etc.*), *K*^+^ and *K*^−^ become unbalanced and the contact line will start moving. For the condensate tail in the cavity, the out-of-balance surface tension force is given by *σ*_sv_ − *σ*_sl_ − *σ* cos *θ*_d_, where *σ*_sv_ is the solid-vapor interfacial tension, *σ*_sl_ is the solid–liquid interfacial tension, *θ*_d_ is the dynamic contact angle (*i.e.*, contact angle associated with the moving contact line) of condensate on the nanostructures inside the cavity. When the difference between the static contact angle *θ*_0_ and the dynamic contact angle *θ*_d_ is not significant, the contact line velocity *u*_c_ can be linearized as (see ESI† for detailed derivation)7
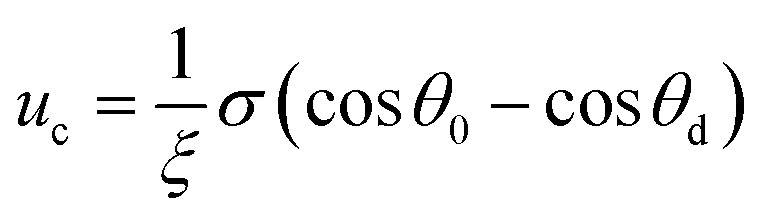
where 
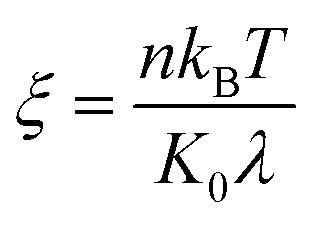
 is the contact line friction coefficient (CLFC) that determines the dissipation rate *ξu*_c_^2^ within the three-phase contact zone,^34^ λ is the jumping step length of liquid molecules and *n* is the number density of the adsorption sites on the surface.

Instead of polyethylene terephthalate,^30^ fluoropolymer-based polytetrafluoroethylene (PTFE) was used as the water repellent coating material on the engineered structures in this work. Little work has been done on contact line friction of water on smooth or rough surfaces coated by fluoropolymer. Therefore we used molecular dynamics (MD) simulation to study the wetting behaviour of water on a PTFE surface in the framework of MKT.^35–38^ For water droplets of tens of nanometers in diameter on a smooth PTFE surface, our MD simulation shows CLFC on the smooth PTFE surface 
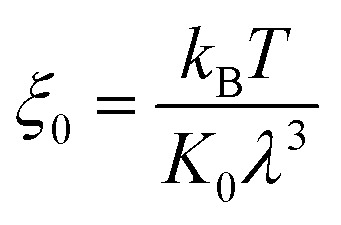
 ∼ 1.2 × 10^−3^ Pa s at 5 °C, which is on the same order of magnitude as the dynamic viscosity of water. For condensate tails entrapped in the first tier cavities, the body of each condensate tail is actually in direct contact with the secondary CNT structures, which is conformally coated by PTFE. Since the condensate tail stays at the Cassie-state with regard to the second-tier nanoroughness, the contact line dissipation is scaled down by a factor of *f*_n_. From Rayleigh dissipation function, the total energy loss due to contact line dissipation in the first tier cavity can be evaluated as:8
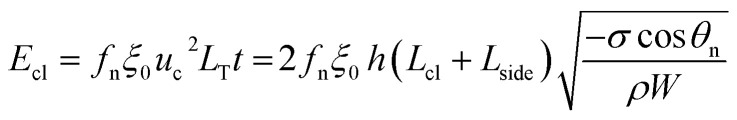
where *L*_T_ = *L*_cl_ + *L*_side_ is the total length of receding contact line of the tail in the PW-Cassie transition, the lateral contact line length *L*_cl_ ∼3.44*r*_p_ (see ESI† for detailed derivation), the vertical contact line length *L*_side_ ∼8 h, *i.e.*, along the surrounding pillar side walls, and *u*_c_ ∼ *U*.

Combining eqn (2), (6) and (8) yields the self-pulling energy barrier of the PW-Cassie transition including adhesion energy *E*_adh_, viscous dissipation *E*_vis_ and contact line dissipation *E*_cl_ in a first tier cavity:9
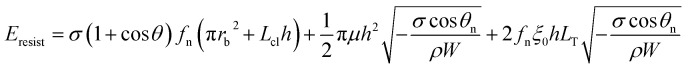


Using pillar height *h* as the characteristic dimension, the nondimensionalized form of eqn (9) becomes:10
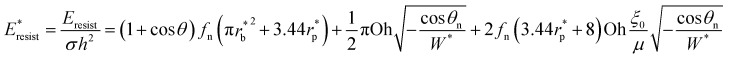


The dimensionless variables are represented with an asterisk, *e.g.*, 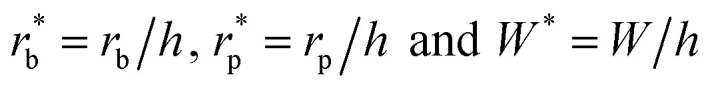. It is noteworthy that the Ohnesorge number Oh is defined as Oh = *μ*/*ρσh* in this analysis. This energy barrier must be overcome by a condensate tail in order to accomplish the PW-Cassie transition *via* self-pulling mode.

### 3.2 Optimization of surface roughness by resistant energy analysis

**Fig. 4:**
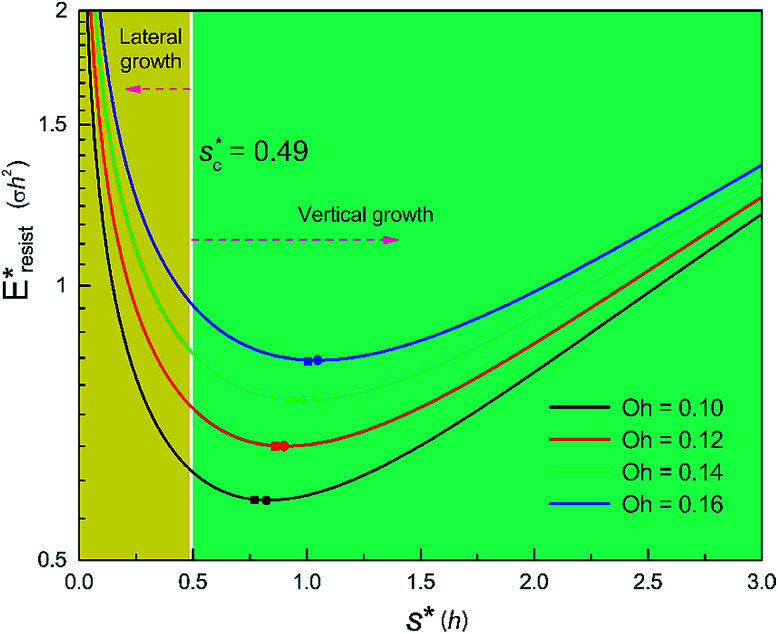
Resistant energy 
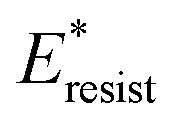
 associated with a condensate tail in a first tier cavity *versus* nondimensional cavity width s* with *f* = 0.08 and Oh be defined as 
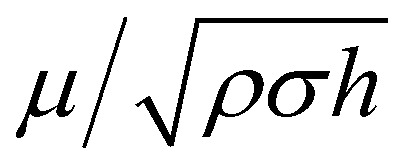
. The critical cavity width 
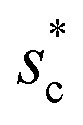
 is 0.49, which is determined by ℛ = 1. Dot on each curve stands for the optimum cavity width 
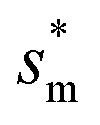
 in each configuration. Square on each curve indicates the primary cavity size where adhesion work equals to viscous dissipation.

We conducted parametric studies to characterize the influence of the first tier pillar geometry on the energy barriers. Fig. 4 shows the variation of resistant energy 
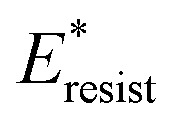
*versus* nondimensional cavity width s* (*i.e.*, s* = *s*/*h* and *s* = *l* − *b*) with the solid fraction *f* = 0.08. It can be seen that for each Oh number there does exist an optimum 
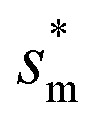
 (and hence optimum pillar width 
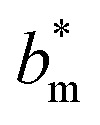
) leading to the minimum resistance.

**Fig. 5:**
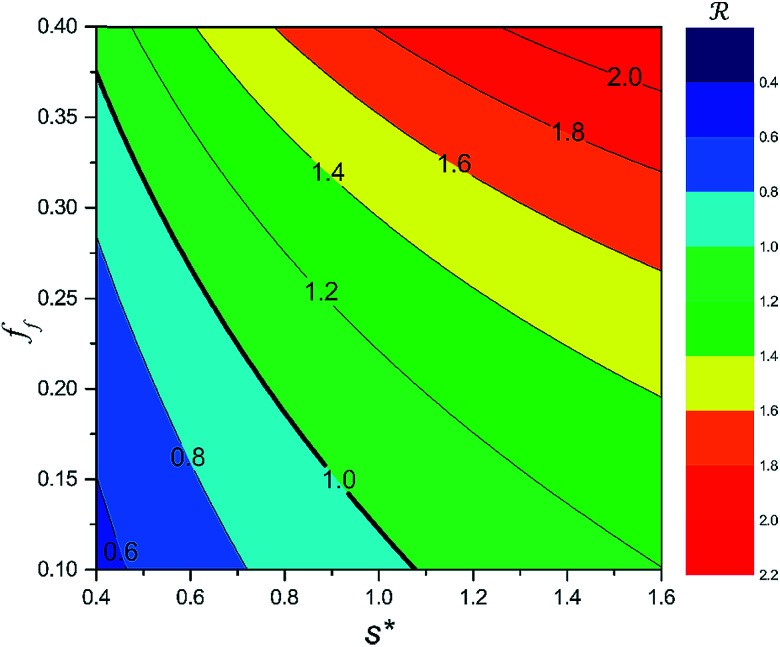
Surface energy change ratio ℛ with respect to cavity width s* and first tier solid fraction *f*_f_ (with 
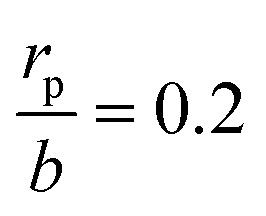
, Oh = 0.10). Area below ℛ = 1 implies vertical growth of condensate droplets becomes energetically favorable.

On the other hand, an ideally-structured condenser surface should be able to promote out-of-plane (vertical) growth of condensate droplets rather than in-plane (lateral) spreading.^10^ We compared surface energy change Δ*E*_vertical_ due to an infinitesimal vertical growth d*z* with surface energy change Δ*E*_lateral_ induced by an infinitesimal lateral growth d*r*. The energy ratio 
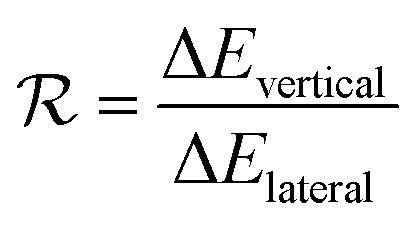
 as a function of cavity width s* and first tier solid fraction *f*_f_ is plotted in Fig. 5 (see ESI† for detailed derivation). For ℛ < 1, the first tier pillars are able to block the lateral wetting of condensates towards the neighbouring cavities, indicative of a thermodynamically favorable configuration of surface structures. The critical cavity width 
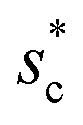
 with ℛ = 1 and the optimum cavity width 
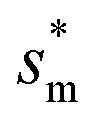
 with minimum resistant energy are shown in Fig. 4 for various Oh (*i.e.*, pillar height *h*) values. It can be seen that for each cavity width larger than the critical width 
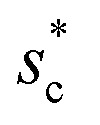
 there does exist an optimum cavity size 
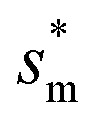
, which not only minimizes resistant energy but also favors vertical growth of condensates. Importantly, by minimizing the resistant energy, the condensate tails formed in the cavities are more apt to the PW-Cassie transition *via* self-pulling mode. It needs to be mentioned that pillar height *h* cannot be arbitrarily short in engineered surface design not only for maintaining proper surface roughness but also for preventing potential sagging of condensate droplets in to the structure cavities even after the PW-Cassie transition.^39^

From Fig. 4, it is clear that for each Oh (hence the height *h* of the pillars) there exists an optimum cavity width 
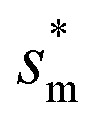
 leading to the lowest resistant energy in a unit cell. Actually, the value of optimum 
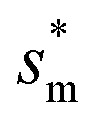
 is constrained by the vertical growth preference factor ℛ and sagging phenomenon^40^ of condensate droplets respectively. The critical cavity width 
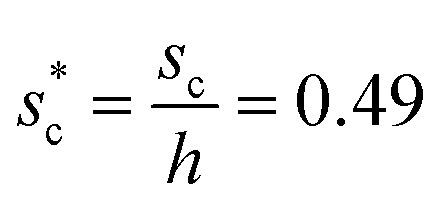
, which is set by ℛ = 1, defines the lower bound for 
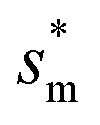
. The upper bound for 
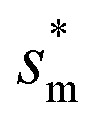
 can be determined by preventing the occurrence of complete sagging, *i.e.*, Cassie to Wenzel transition, of the condensate droplet after the PW-Cassie transition. The critical pillar height *h*_sag_ for the occurrence of complete or full sagging of a droplet sitting on top of pillars is (see ESI†)11
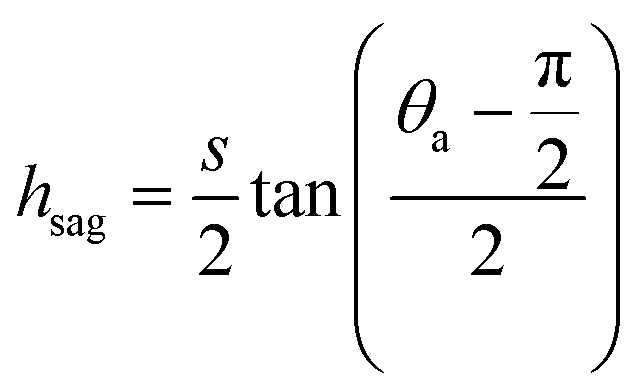
where *θ*_a_ is the advancing contact angle of droplets on the pillar sidewalls, which is covered by the second tier roughness. Therefore, the critical cavity width 
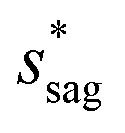
 preventing complete sagging occurrence becomes12
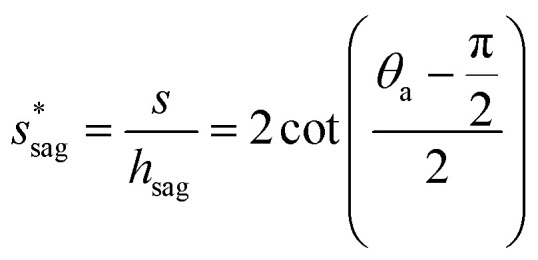


Assuming *θ*_a_ ≅ *θ*_n_ ≅ 150° on the CNTs with *f*_n_ = 0.25, the upper limit of cavity width bounded by sagging occurrence on the textured surface is 
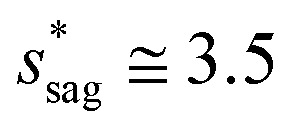
.

**Fig. 6:**
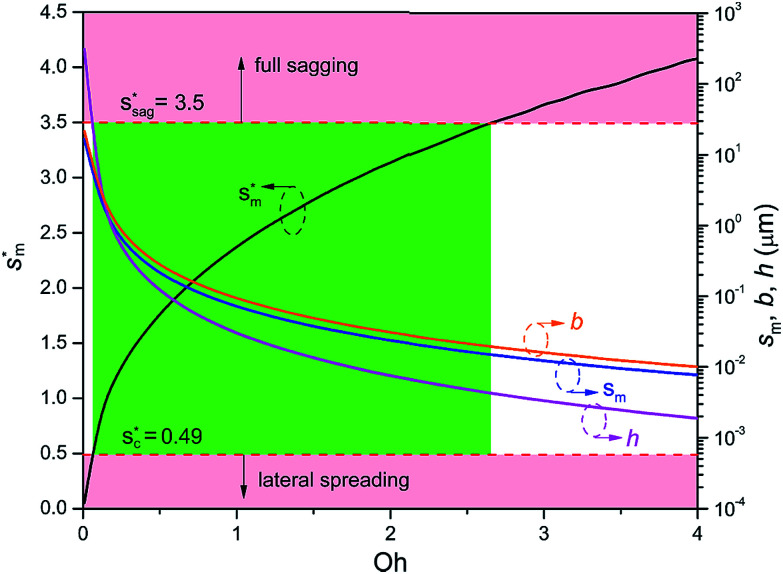
The nondimensional optimum cavity width 
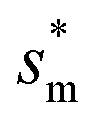
, pillar height *h*, pillar width *b* and optimum cavity width *s*_m_ with respect to 
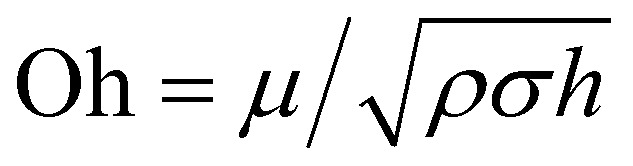
. The geometries and configuration of primary roughness in the green regime can be adopted in condenser design for facilitating continuous dropwise condensation.

**Fig. 7:**
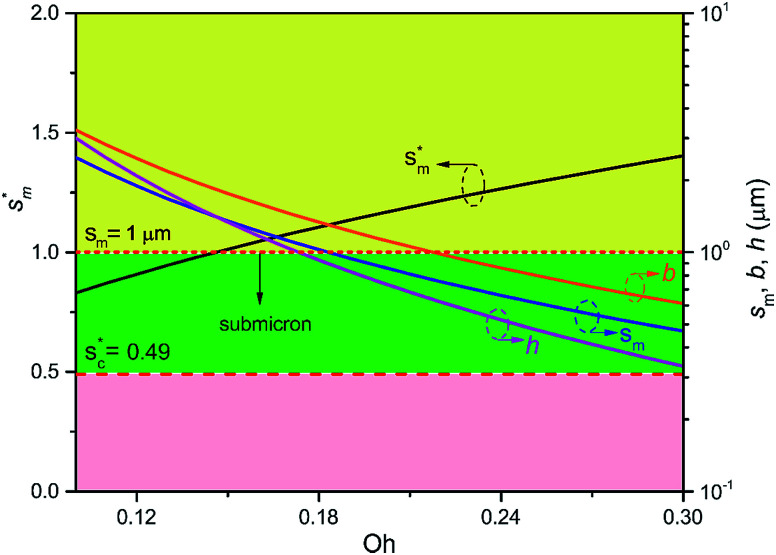
The nondimensional optimum cavity width 
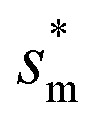
, pillar height *h*, pillar width *b* and optimum cavity width *s*_m_ with respect to the Oh number in the range of 0.1–0.3.

Fig. 6 shows the effects of Oh on the nondimensional optimum cavity width 
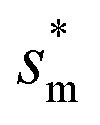
, dimensional optimum cavity width *s*_m_, the pillar height *h* and pillar width *b* of the primary roughness. As Oh approaches to 0.035, indicative of a pillar height of ∼15 μm, the optimum 
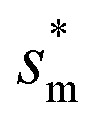
 reaches the critical lower bound 
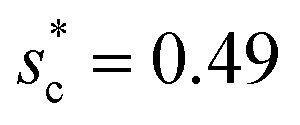
, beyond which the surface structures cannot effectively prevent the undesired lateral spreading. As Oh increases, the nondimensional optimum 
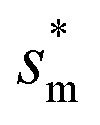
 increases whereas the optimum cavity width *s*_m_, pillar height *h* and pillar width *b* eventually decrease to the submicron levels, respectively. When Oh approaches to 2.7, indicative of the pillar height as low as tens of nanometers, the optimum 
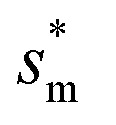
 gets close to the upper bound 
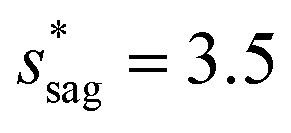
. But between the lower and the upper limits for 
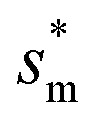
, the shorter the height of the pillar (and hence the narrower the width of the cavity), the sooner the cavity is filled up with condensate and the sooner the self-pulling stage can be achieved. Fig. 7 shows a portion of the Fig. 6 for Oh in the range of 0.1–0.3. The 1 μm line for *s*_m_, *b* and *h* is displayed to demarcate the micron and submicron regimes. As Oh increases, the values of *s*_m_, *b* and *h* decrease as shown in Fig. 6 as well. For Oh > 0.2, the values of *s*_m_, *b* and *h* enter the submicron regime. Therefore, having both tiers of roughness in the submicron scale and also designing the first tier structure to match the optimum value shown in Fig. 4 (there exists an optimum value for each Oh, and hence for each pillar height *h*) can facilitate the PW condensate removal as a result of the remarkably alleviated resistant energy. On the other hand, the design of such structured surfaces with both the two tier roughnesses in submicron scale also imposes a limitation on how small the first tier can be, which is beyond the scope of this work.

**Fig. 8:**
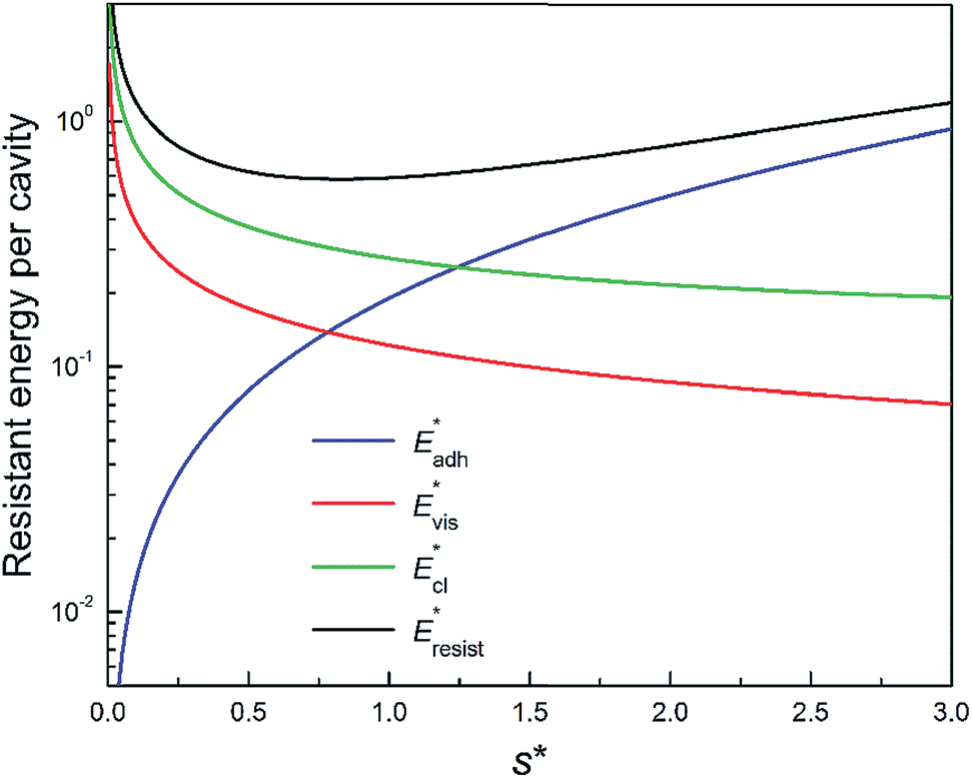
Variation of the nondimensionalized viscous dissipation 
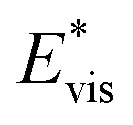
, adhesion work 
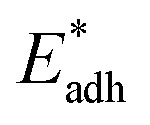
, contact line dissipation 
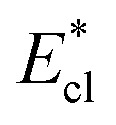
 and resistant energy 
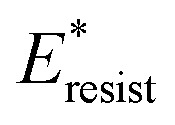
 in a cavity with roughness spacing s* (*f* = 0.08 and Oh = 0.10).

We further compared in Fig. 8 the evolution of viscous dissipation 
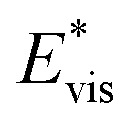
, adhesion work 
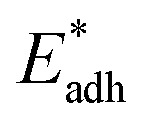
, contact line dissipation 
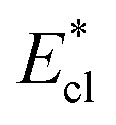
 and resistant energy 
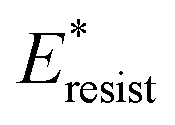
 with pillar gap s* while *f* = 0.08 and Oh = 0.1. Same as resistant energy 
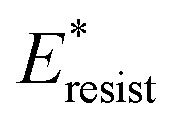
, the above energy factors are nondimensionalized by *σh*^2^. Increasing pillar gap s* has a prominent mitigating effect on viscous dissipation and contact line dissipation as opposed to an intensified effect on adhesion. As discussed above, there exists an optimum cavity size 
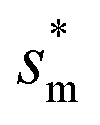
 giving rise to the minimum resistant energy 
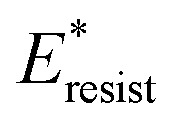
. It is noteworthy that for 
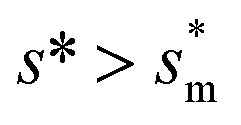
 the work of adhesion, viscous dissipation and contact line dissipation are all prominent, but for 
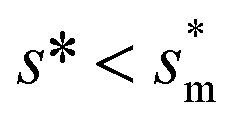
 the resistance is dominated by viscous dissipation and contact line dissipation.

### 3.3 Condensate spatial density influence on resistant energy

The topography of a condenser surface has significant influence on nucleation site density as nucleation sites generally increase with the decrease of the characteristic size of surface roughness. Nevertheless, the importance of controlling nucleation density in pursuit of CDC could be evidently illustrated by selecting different hypothetical condensate spatial densities, indicative of the influence of surface structure density on resistant energy. In this analysis, condensate site on a two tier rough surface refers to a first tier cavity with a forming PW condensate entrapped therein after the initial nourishing process of a nucleate embryo.

Discrete dropwise condensates should be methodically maintained while the uncontrolled lateral spreading should be avoided or at least delayed during condensate growth in surface cavities, otherwise dropwise condensation may not continue and the surface would eventually get flooded. To satisfy this criterion the spacing between the condensate sites *L* should be at least 2 times the roughness period or spacing *l*, *i.e.*, 
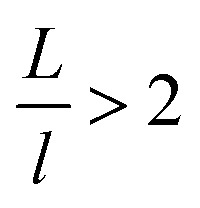
.^41^ The spacing between condensate sites could be given by 
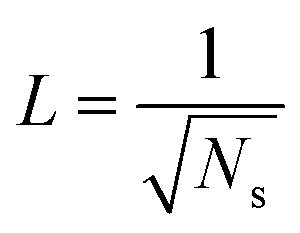
, where *N*_s_ denotes the density of condensate droplets. Then the above site criterion can be rewritten as13
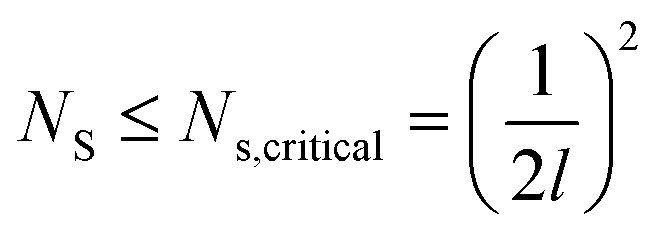


**Fig. 9:**
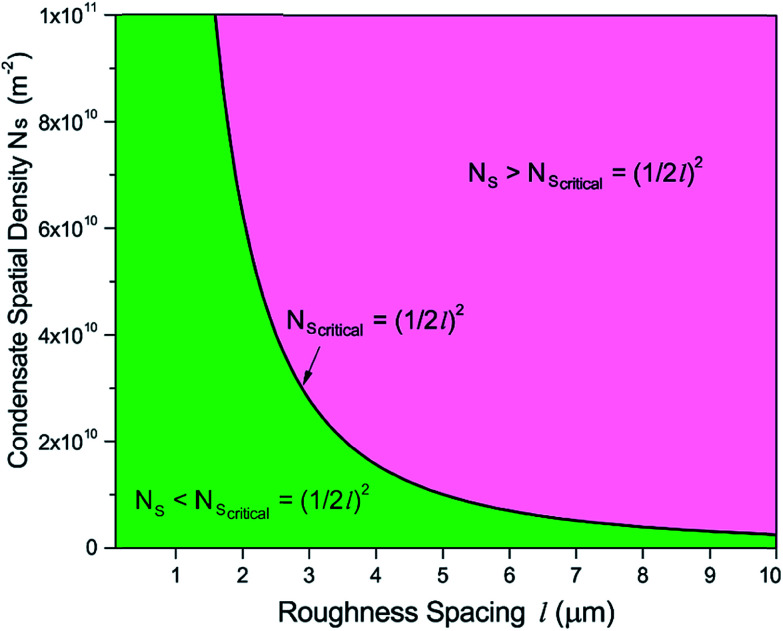
Critical condensate site density *N*_s,critical_ for different roughness period *l*.

Fig. 9 shows the critical values of condensate sites *versus* roughness spacing *l* (eqn (13)). The region beneath the critical curve (green area) represents the condensate site densities satisfying dropwise condensation criterion mentioned above. For *N*_s_ values above the critical curve (red area) multiple condensates may form around a unit cavity. As these condensates grow, they would merge with those in neighbouring cavities leading to film condensation and may eventually flood the surface. Regarding one of our two-tier superhydrophobic surfaces with *l*_0_ = 9 μm, the benchmark site criterion is 
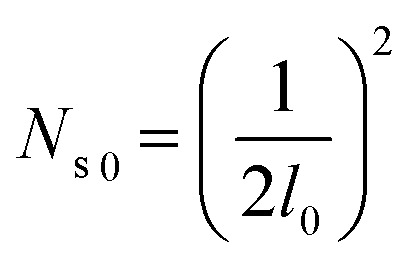
. In a general case with varying roughness spacing *l*, the critical condensate site density *N*_s,critical_ can be related to *N*_s_0__ as14
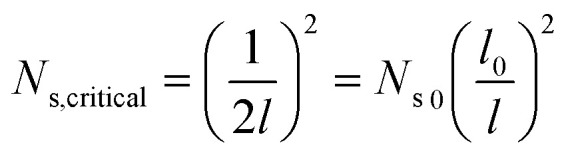


To guarantee *N*_s_ falls beneath the critical curve in Fig. 9, the following criterion regarding the power *n* of 
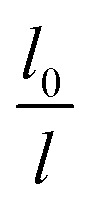
 must be met15
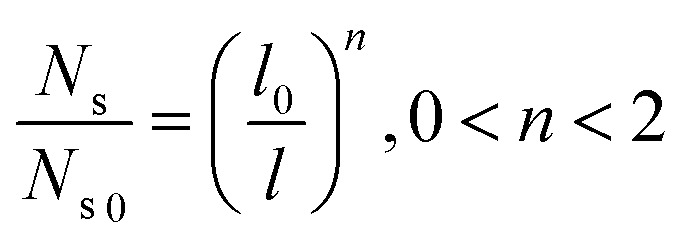


**Fig. 10:**
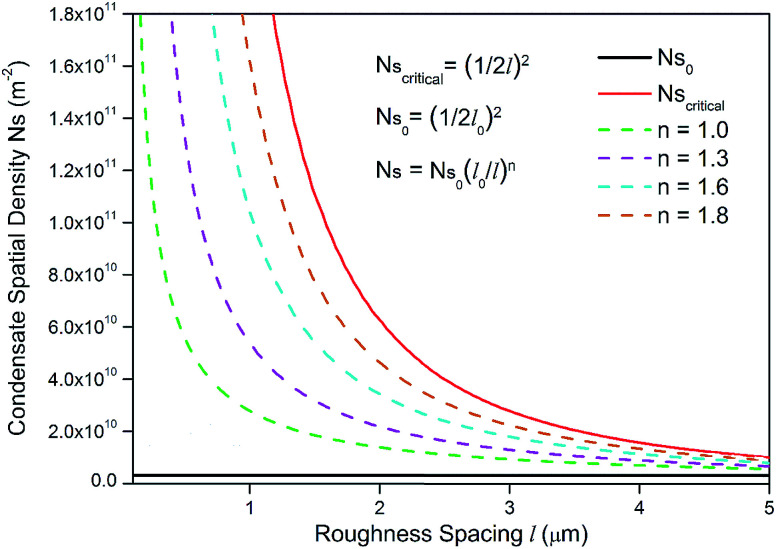
Different hypothetical condensate site density *N*_s_*versus* roughness spacing *l*.

As shown in Fig. 10, the power *n* of 
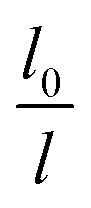
 was chosen to be 1, 1.3, 1.6, and 1.8 respectively to make *N*_s_ remain below the critical curve, *i.e.*, in the CDC region. These condensate site density curves are hypothetically chosen in order to provide an insight into the effect of condensate site density on the overall resistant energy of surfaces of different roughness characteristics.

**Fig. 11:**
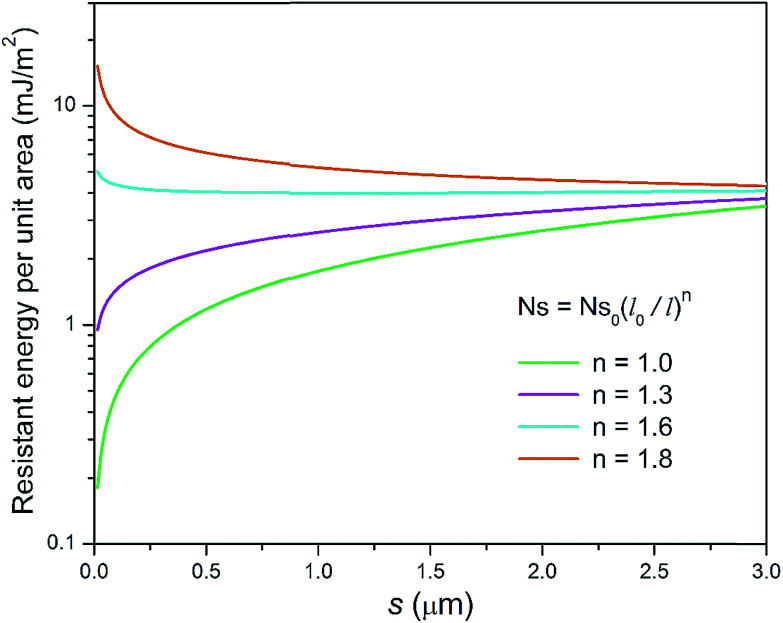
Resistant energy per unit area *versus* primary cavity size *s* for different hypothetical condensate site densities.

It can be seen in Fig. 11 that for different condensate site densities the overall resistant energy of the surface displays various behaviours as the primary cavity size *s* decreases. When the condensate site density is maintained at a moderate level (*n* = 1 or 1.3), the resistant energy of the surface is approximately in proportion to *s*. In contrast, as the condensate site density rises to even higher levels (*n* = 1.6 or 1.8), the overall resistant energy of the surface could increase especially for smaller roughness sizes, despite the resistant energy of a unit cell is decreasing (also see ESI†). In other words, the significant increase in the number of condensate sites leads to more condensate tails formed in the cavities of first-tier structures. Therefore, the overall resistant energy rises as a result of the increase of condensate sites. From the experimental point of view, to maintain condensate sites within a proper range (*n* < 1.6), superbiphilic surfaces formed by lithographically patterning superhydrophilic islands on superhydrophobic surfaces^42^ or chemical micropatterns^43^ can be employed for spatial control of at least microscale droplets during condensation, which is a new research theme in dropwise condensation on engineered surfaces.

## 4. Discussion

Even though it has been a challenge to realize sustained CDC on engineered surfaces in real applications, there have recently been reported experimental works demonstrating jumping-droplet-enhanced condensation on nanostructured surfaces in carefully-designed vapor chambers.^8,24,44–46^ Instead of using conventional wicks, Boreyko and Chen developed a novel vapor chamber with jumping-drop liquid return,^44^ in which superhydrophobic condenser is composed of 150–300 nm clusters of 50–100 nm silver nanoparticles on copper, and the overall lumped heat transfer coefficient has reached as high as 55 kW m^−2^ K^−1^. Both tiers of silver nanoparticles structures on the condenser are in nanoscale, which is in excellent agreement with our analysis of structural optimization. Two other recent works, which used superhydrophobic Si nanowires^45^ and hydrophobic Cu nanowires^46^ respectively on their condensers, have confirmed enhanced condensation heat transfer due to droplet jumping behaviour. It is noteworthy that these nominal single layer nanostructures actually consist of bundles of nanowires with microscale gaps, indicative of essentially two-tier hierarchical structures. Even though our resistant energy analysis starts with two-tier structures, it can be easily extended to surfaces with nominal one-tier nanotextures,^8^ which may be essentially composed of dual-scale roughness as more obvious in the clustered ribbed-nanoneedle structures.^24^ Due to the irregular and nonuniform geometries of surface roughness present in these works, we could only conduct a rough comparison of these experimental studies with our theoretical analysis. Based on the major features or characteristic lengths extracted from the surface structures of the abovementioned works, the structural comparison in the form of scattered points is shown in Fig. S8.† It can be seen that most of these surface configurations satisfy the continuous dropwise condensation criteria, which is illustrated by the central green region, proposed by us. In the condensation experiment by Yang *et al.*,^46^ the nanofibers are ∼20–30 μm tall with a high aspect ratio so that condensate droplets may be initially formed in the Cassie state instead of in the partial wetting (PW) morphology, which is not in accord with the situation of the PW-Cassie transition as discussed in our analysis.

**Fig. 12:**
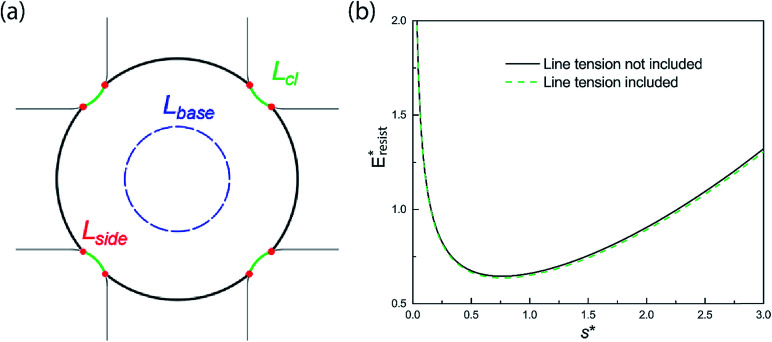
(a) Top view of line tension distribution of a PW droplet. The red dots indicate the eight vertical segments along the first tier pillars. (b) Effect of line tension on the resistant energy for Oh = 0.10.

On the other hand, when the surface roughness approaches nanoscale, the intrinsic line tension effect^47^ may play a certain role in the PW-Cassie transition. For a PW droplet anchored in the surface cavities, the line tension occurs at the three-phase contact line and its distribution is illustrated in Fig. 12(a). The resistant energy *E*_lt_ caused by line tension can be calculated by:

16 *E*_lt_ = *σ*_κ_(*L*_cl_ + *L*_base_ + *L*_side_) ≈ *σ*_κ_(*L*_cl_ + 2π*r*_b_ + 8 h)

where *σ*_κ_ is the line tension coefficient and *L*_base_ is the contact length on the cavity valley. The value of line tension coefficient *σ*_κ_ was estimated in our MD simulation by comparing the difference between the apparent contact angle and the contact angle predicted by the Young's equation^47^

17 *σ*_κ_ = (*σ*_sv_ − *σ*_sl_ − *σ* cos *θ*_0_)*κ*

where *κ* is the surface curvature of liquid, and here *θ*_0_ refers to the static contact angle of nanodroplets obtained by MD simulations. The positive value of *σ*_κ_ enhances surface hydrophobicity, whereas the negative value promotes surface hydrophilicity. The calculation of each component of the interfacial tensions followed the standard procedures suggested by a previous study.^48^ And the line tension coefficient *σ*_κ_ for water spreading on fluorinated surfaces was calculated to be ∼2 × 10^−10^ J m^−1^. As shown in Fig. 12(b), the difference between the total resistant energy, which takes the line tension into consideration, and the resistant energy excluding the line tension effect is negligible for Oh = 0.10.

We also studied the relative strength *e*_E_ of line tension in the resistant energy associated with the PW-Cassie transition, which is defined as18
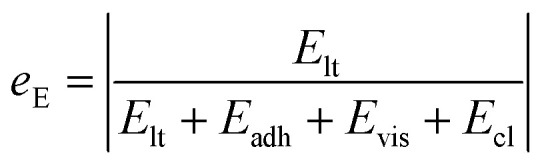


And the values of *e*_E_ for different Ohnesorge numbers are shown in Table 1.

**Table 1:** Values of *e*_E_ for different Ohnesorge numbers

Oh	0.10	0.12	0.14	0.16
*e*_E_	1.16%	1.36%	1.53%	1.68%

Therefore, according to our analysis, line tension is not a dominant factor in resisting PW-Cassie transition at least in nanoscale roughness.

## 5. Conclusions

Continuous and sustained dropwise condensation on engineered surfaces places stringent requirements on careful design of surface structure length scale and geometry, as well as meticulous control of condensate morphology and spatial distribution.^49–51^ We carried out optimization of two-tier structured surfaces by minimizing the resistant energy that impedes the PW-Cassie transition. Our analysis of condensate growth in a first tier cavity shows that there does exist an optimum first tier geometry that can tremendously mitigate the resistant energy while allowing condensates to preferentially grow in the out-of-plane direction. We further showed that reducing surface roughness to submicron scale could be promising in achieving sustainable dropwise condensation due to the even lower resistance to the PW-Cassie transition. In addition to playing an important role in confining and controlling embryo formation, the second-tier nanotextures can effectively mitigate contact line dissipation and contact line pinning of the condensates. On the overall condenser surface, discrete condensate distribution is desired for delaying condensate flooding. Due to the relatively smaller energy barrier (Gibbs free energy) in the cavities of first tier structures, most of the condensate droplets in the PW state can be discretely confined therein. In this respect, superhydrophobic condenser surfaces with multiscale structures are superior to flat or solely nanotextured surfaces in controlling condensate spatial distribution.^52,53^ In order to further meticulously and precisely control condensate droplet density at elevated supersaturation, superbiphilic surfaces formed by lithographically registering superhydrophilic spots on superhydrophobic surfaces can be developed as novel condensers in order to achieve CDC. This energy-based analysis can help us design engineered surfaces that can sustain CDC in strong condensation with elevated supersaturation and consequently give rise to enhanced condensation heat transfer.

## Authors' contributions

J. C. and A. V. conceived the concepts of this research. L. Z. obtained contact line friction coefficient by MD simulation. All authors contributed to finalizing this manuscript.

## Conflicts of interest

The authors declare no competing financial interests.

## Supplementary Material

NA-001-C8NA00237A-s001
