# Supplementary material for: Resistant energy analysis of self-pulling process during dropwise condensation on superhydrophobic surfaces
Source: Nanoscale Adv. 2018 Dec 20;1(3):1136–47. doi: 10.1039/c8na00237a (PMC9473257; doi:10.1039/c8na00237a)
Supplement: NA-001-C8NA00237A-s001 [file NA-001-C8NA00237A-s001.pdf]

Electronic Supplementary Material (ESI) for Nanoscale Advances.
This journal is © The Royal Society of Chemistry 2018

Supplemental Materials

Resistant Energy Analysis of Self-Pulling Process during Dropwise Condensation on Superhydrophobic Surfaces

Aref Vandadi,[a] Lei Zhao,[b] Jiangtao Cheng[a,b]*

[a]Department of Mechanical and Energy Engineering, University of North Texas, Denton, TX 76207, USA

[b]Department of Mechanical Engineering, Virginia Polytechnic Institute and State University, Blacksburg, VA 24061, USA

*Email: chengjt@vt.edu

I. Derivation of contact line length

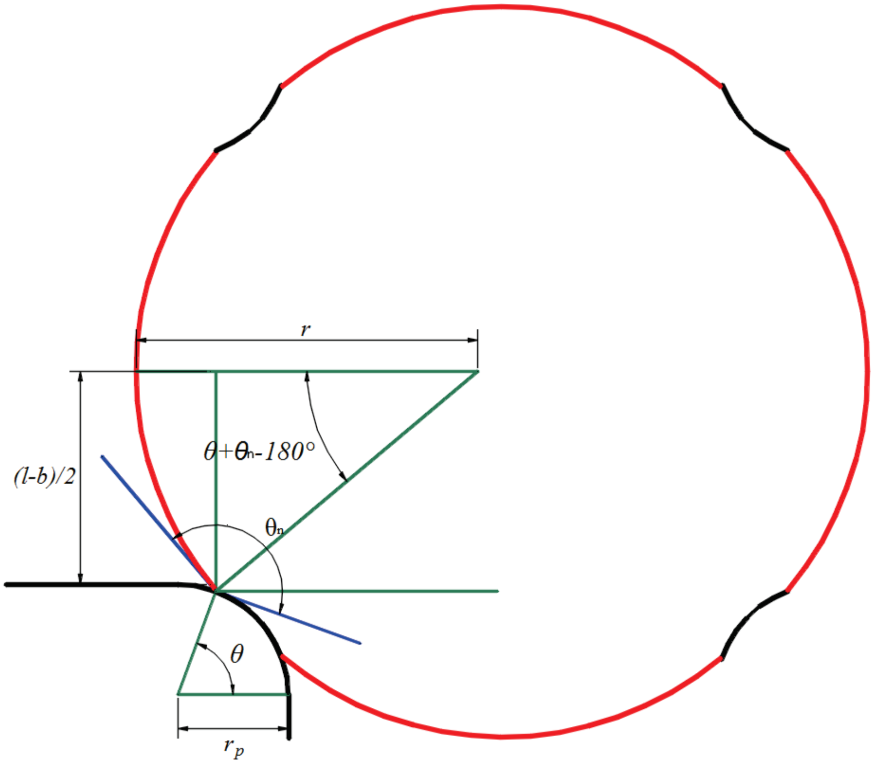

Figure S1. Illustration of contact line (top view)

As illustrated in Figure S1, the contact line consists of two parts: the solid-liquid interface L_{cl} (marked as dark lines) and the liquid-vapor interface L_{LV} (marked as red lines). The length of each part is subjected to the position where L_{cl} and L_{LV} intersect, which can be is a function of the central angle θ.

$$L_{cl} = l_{SL}(\theta) \text{ and } L_{LV} = l_{LV}(\theta) \tag{1}$$

Assuming the solid-fluid interface l_{SL} follows a circular shape, its radius is calculated to be

$$r = \frac{0.5(l-b) + r_p(1-\sin\theta)}{\sin(\theta+\theta_n-\pi)} \tag{2}$$

$$\frac{r}{l} = \frac{0.5\left(1-\sqrt{f_f}\right) + \frac{r_p}{l}\sqrt{f_f}(1-\sin\theta)}{\sin(\theta+\theta_n-\pi)} \tag{3}$$

From geometrical constraints, the total length of L_{SL} and L_{LV} is calculated to be

$$L_{cl} = 8\left(\theta-\frac{\pi}{4}\right)r_p \tag{4}$$

$$L_{LV} = 8(\theta+\theta_n-\pi)r \tag{5}$$

Since r_p is relatively small comparing to the pillar spacing l, we can assume that the total length of interfaces (including solid-liquid interface and liquid-vapor interface) is constant. In this scenario, the total surface free energy of the system can be calculated as

$$E_{tot} = \gamma_{LV} L_{LV} + L_{cl}(\gamma_{SL} - \gamma_{SV} - \gamma_{LV}) \tag{6}$$

where γ is the interfacial intension and the subscripts of L, V and S represent liquid phase, vapor phase and solid phase respectively. Combining Young's equation, E_{tot} can be rewritten as

$$E_{tot} = \gamma_{LV} L_{LV} - \gamma_{LV} L_{cl}(1 + \cos\theta_n) \tag{7}$$

$$\begin{aligned} \frac{E_{tot}}{\gamma_{LV} l} = 8(\theta + \theta_n - \pi) \frac{0.5\left(1 - \sqrt{f_f}\right) + 0.2\sqrt{f_f}(1 - \sin\theta)}{\sin(\theta + \theta_n - \pi)} \\ - \frac{8r_p}{l}\left(\theta - \frac{\pi}{4}\right)(1 + \cos\theta_n)\sqrt{f_f} \end{aligned} \tag{8}$$

A stable system must be maintained at its minimal free energy. In this study, the central angle must satisfy $\frac{\pi}{4} \leq \theta \leq \frac{\pi}{2}$. The condition of $\frac{\partial E_{tot}}{\partial \theta} = 0$ provides that the central angle θ is the function of f_f. The value of θ can be solved numerically.

Under the condition of $\theta_n \approx 150°, f = 0.08, r_p = 0.2b$,

$$\theta = 69.61°, L_{cl} = 3.44 r_p \tag{9}$$

II. Molecular Kinetic Theory

The basic idea underlying the MKT is that the macroscopic three-phase contact line motion can be viewed as the statistical results of water particles' adsorption to and detachment from adsorption sites on a surface[1-4], as illustrated in Figure S2. The adsorption and detachment movements are controlled by the outward frequency K^+ and inward frequency K^- respectively.

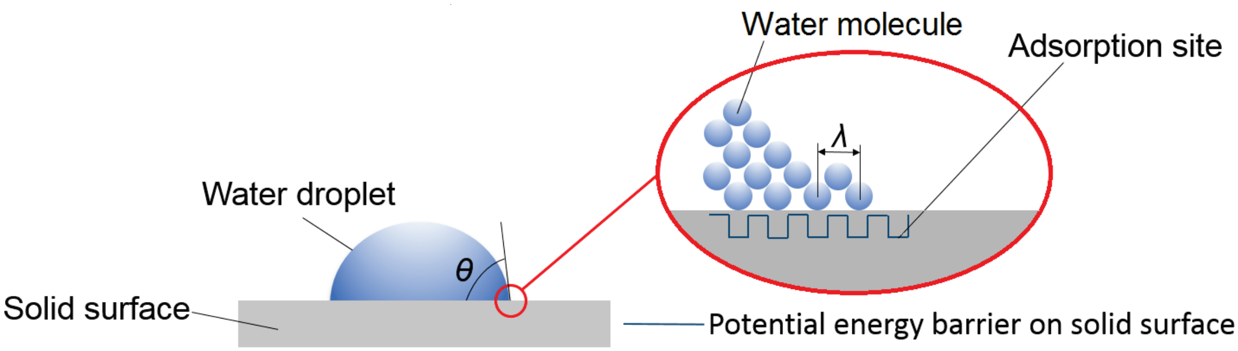

Figure S2. Schematic of the MKT with energy barriers illustrated. Adsorption sites are shown as cavities in this figure.

$$u_c = \lambda(K^+ - K^-) \tag{10}$$

$$K^+ = \frac{k_B T}{h} \exp(-\frac{\Delta G}{N_A k_B T}) \exp\left(\frac{\sigma(\cos\theta_0 - \cos\theta)\lambda^2}{2k_B T}\right) \tag{11}$$

$$K^- = \frac{k_B T}{h} \exp(-\frac{\Delta G}{N_A k_B T}) \exp\left(-\frac{\sigma(\cos\theta_0 - \cos\theta)\lambda^2}{2k_B T}\right) \tag{12}$$

where u_c is the contact line velocity (wetting velocity), λ represents the unit displacement length and *ΔG* is the molar free energy, defined as the energy barrier that water molecules need to overcome to reach the activated state (so as to either advance or retreat), and is one of the key parameters in the MKT framework. The other parameters included in above equations are the Boltzmann's constant k_B, temperature *T*, the Planck's constant *h* and the Avogadro constant N_A. At equilibrium state where the dynamic contact angle θ reaches the static contact angle θ_0, both the adsorption frequency and detachment frequency equal to a constant value K_0

$$K^+ = K^- = K_0 = \frac{k_B T}{h} \exp(-\frac{\Delta \mathrm{G}}{N_A k_B T}) \tag{13}$$

In the MKT framework, the surface free energy is considered to be dissipated mainly in the form of molecular retarding, or molecular friction. From Rayleigh dissipation function[5], the molecular friction coefficient can be calculated to be:

$$\xi = \frac{\sigma(\cos\theta_0 - \cos\theta)}{u_c} \tag{14}$$

Also if $\sigma\lambda^2(\cos\theta_0 - \cos\theta) \ll 2k_B T$, the molecular friction coefficient can be obtained as

$$u_c \approx \frac{K_0 \sigma \lambda^3 (\cos\theta_0 - \cos\theta)}{k_B T} \tag{15}$$

$$\xi = \frac{k_B T}{K_0 \lambda^3} \tag{16}$$

III. Energy barriers

A droplet formed in the cavity of first tier is pinned by surrounding pillars and can grow in its lateral direction or vertical direction. We evaluate the growing direction of the droplet by the corresponding energy barriers in an incremental increase in the lateral or vertical direction.

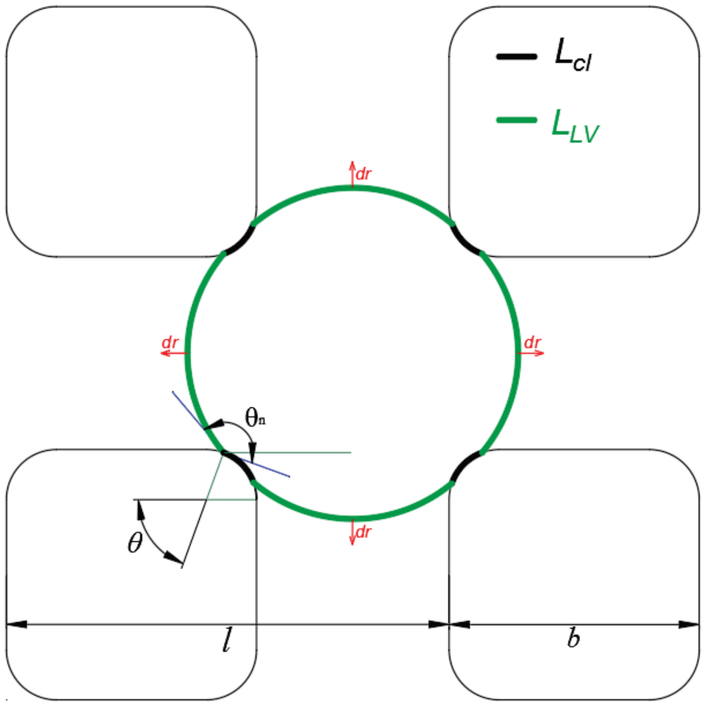

Figure S3 Illustration of lateral growth of droplet trapped in a single cell

Figure S3 illustrates the lateral growth of droplet. The energy barrier $\Delta E_{lateral}$ that the droplet for the droplet to overcome to grow is can be divided into three parts, ΔE_{l1}, ΔE_{l2}, and ΔE_{l3}.

ΔE_{l1} refers to the energy barrier induced by the spreading of the droplet base.

$$\Delta E_{l1} = (\sigma_{SL} - \sigma_{SV}) L_{LV} dr = -\sigma \cos\theta_n \, L_{LV} dr \tag{17}$$

ΔE_{l2} is equal to the energy resulting from the lateral growth of droplet top.

$$\Delta E_{l2} = \sigma L_{LV} dr \tag{18}$$

ΔE_{l2} roots in the lateral advancement of the contact line on pillar sidewalls.

$$\Delta E_{l3} = \frac{8h(\sigma_{SL} - \sigma_{SV})dr}{\sin\theta} = -\frac{8h\sigma\cos\theta_n}{\sin\theta} dr \tag{19}$$

Conclusively,

$$\Delta E_{lateral} = \sigma L_{LV}(1 - \cos\theta_n) dr - \frac{8h\sigma\cos\theta_n}{\sin\theta} dr \tag{20}$$

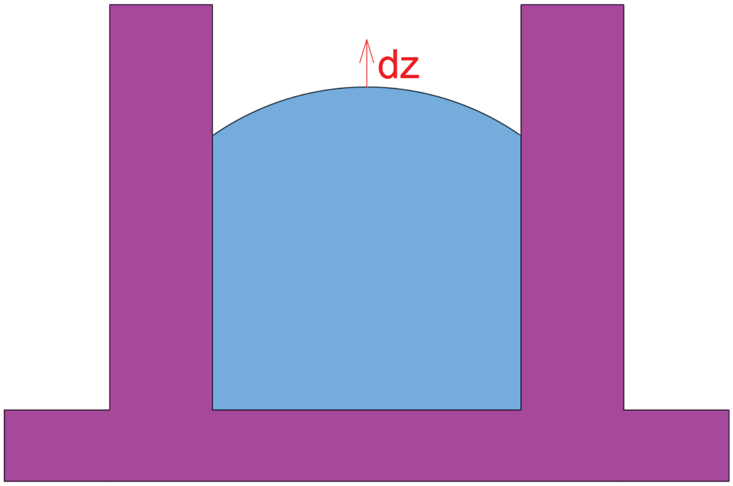

Figure S4. Illustration of vertical growth of droplet trapped in a single cell

The vertical growth of droplet is shown in Figure S4. The energy barrier $\Delta E_{vertical}$ can be divided into two parts, ΔE_{v-cl} and ΔE_{v-LV}.

ΔE_{v-cl} results from the spreading of contact line in vertical direction.

$$\Delta E_{v-cl} = (\sigma_{SL} - \sigma_{SV})L_{cl}dz = -\sigma L_{cl} \cos\theta_n \, dz \quad (21)$$

ΔE_{v-LV} is induced by the growth of liquid-vapor interface in vertical direction.

$$\Delta E_{v-LV} = \sigma L_{LV} dz \quad (22)$$

The energy barrier for vertical growth can be calculated to be

$$\Delta E_{vertical} = -\sigma L_{cl} \cos\theta_n \, dz + \sigma L_{LV} dz \quad (23)$$

The value of L_{cl} and L_{LV} can be solved by following the procedures in section S1. By putting the volume changes required for vertical and lateral growth equal, we get

$$\frac{dz}{dr} = \frac{h(L_{cl} + L_{LV})}{4r^2[\theta + \theta_n - \pi - \sin(\theta + \theta_n - \pi)] + \left[l - b + 2r_p(1 - \cos\theta)\right]^2 - 4r_p^2\left[\theta - \frac{\pi}{4} - \cos\theta\,(\sin\theta - \cos\theta)\right]} \quad (24)$$

Therefore, the ratio of energy barrier due to vertical growth and lateral growth is defined as

$$\mathcal{R} = \frac{\Delta E_{vertical}}{\Delta E_{lateral}} \quad (25)$$

$\mathcal{R} = \frac{\Delta E_{vertical}}{\Delta E_{lateral}}$ is an implicit function of f_f, b, h, r_p, θ_n and can be solved by numerically solving above equations.

IV. Time for tail rise in our analysis vs. capillary time

In our time analysis of tail rise, we have

$$F \approx -\pi\sigma W \cos\theta_n, \quad m \approx \rho\pi W^2 h/4, \quad U^2 = \frac{Fh}{m} \tag{26}$$

So time for tail rise can be scaled as

$$\tau = \frac{h}{U} = \frac{1}{2}\sqrt{\frac{\rho W h^2}{-\sigma\cos\theta_n}} \approx \sqrt{\frac{\rho r h^2}{\sigma}} \qquad (W \approx r) \tag{27}$$

Capillary time is as follows which has the same scale as time $\boldsymbol{\tau}$ in our analysis of tail rise:

$$t_c = \sqrt{\frac{\rho r^3}{\sigma}} \approx \sqrt{\frac{\rho r h^2}{\sigma}} \approx \tau \tag{28}$$

V. Droplet growth on the smooth hydrophobic surface

The following shows the snapshots for dynamic growth of droplets on smooth hydrophobic surface.

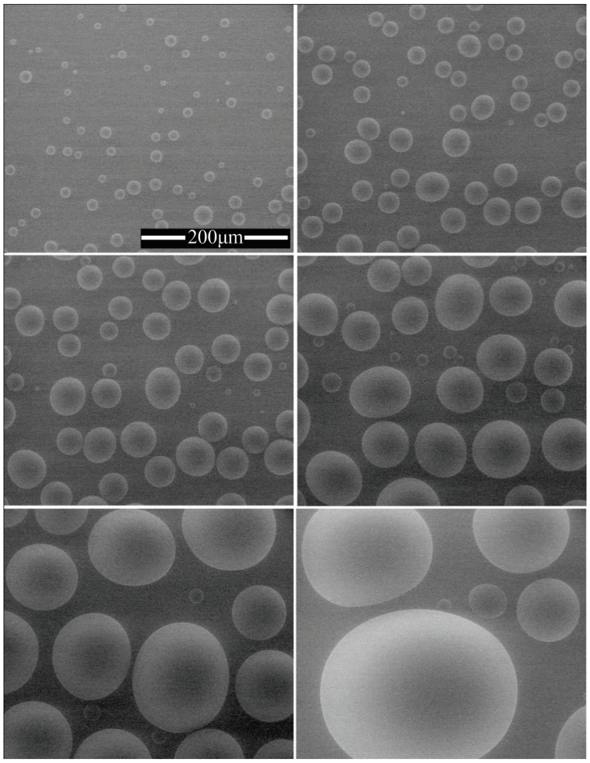

Figure S5. Dynamic growth of droplets on smooth hydrophobic surface

We have used hydraulic diameter to measure the diameter of the non-spherical droplets.

$$D_H = \frac{4A}{P} \tag{29}$$

VI. The critical *h* for the occurrence of full sagging

Upon full or complete sagging, the meniscus underneath a droplet sitting on top of pillars touches the base of the cavity and results in the transition from the Cassie state to the Wenzel state.

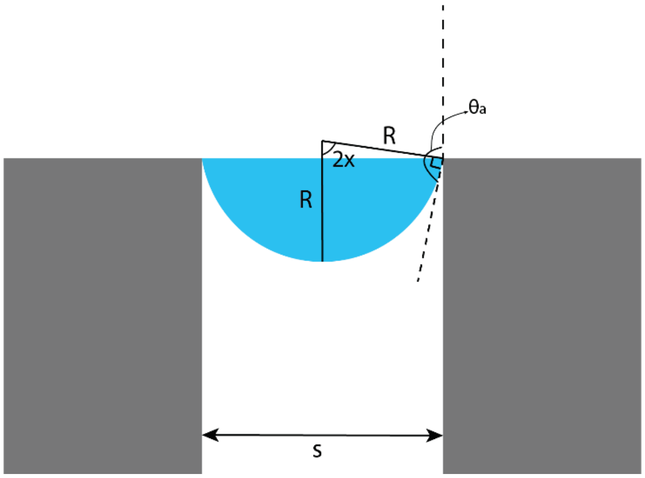

Figure S6. Critical *h* for the occurrence of full sagging

The critical h for which full sagging occurs can be calculated as follows,

$$h_{sag} = R - R\cos 2x \tag{30}$$

$$s = 2R\sin 2x \tag{31}$$

Therefore h_{sag} can be written as,

$$h_{sag} = \frac{s}{2}\frac{1-\cos 2x}{\sin 2x} \tag{32}$$

Using $\cos 2x = \cos^2 x - \sin^2 x$ and $\sin 2x = 2\sin x\cos x$, the critical h for complete sagging occurrence can be written as,

$$h_{sag} = \frac{s}{2}\tan(\frac{\theta_a - {}^{\pi}/_{2}}{2}) \tag{33}$$

VII. The effect of nucleation site density on overall resistant energy

We have fixed the height of the pillar to figure out the optimum pillar size that minimizes the resistant energy of the cell. If the height of the pillar is also lowered in accordance with the width of the pillar, it is expected to see a decreasing resistant energy for a single cell as shown in Figure S7.

It is shown in the main manuscript (Figure 11) that even though the energy of the single cell decreases as the pillar size is lowered (for constant *h/b* ratios), the overall energy of the surface can increase as the nucleation density goes up. It underlines the importance of controlling the number of the nucleation sites during condensation.

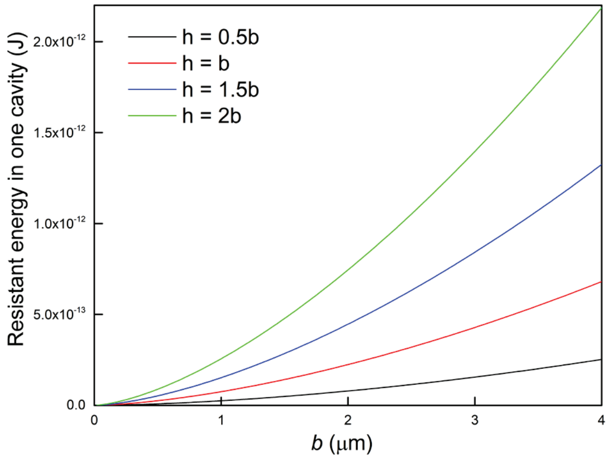

Figure S7. Resistant energy of a cell for different *h*/*b* ratios

VIII. Comparison of our resistant energy analysis with available experimental works

Some experimental works of dropwise condensation on engineered surfaces as listed in Refs 8, 24, 44, 45 and 46 in our manuscript have demonstrated enhanced heat transfer in carefully designed vapor chambers. All these experimental works were carried out on superhydrophobic surfaces with nanofibers/wires or clusters of nanoparticles and have demonstrated competitive advantages over traditional condensers on flat surfaces. Due to the irregular and nonuniform geometries of surface roughness present in these works, we could only conduct a rough comparison of our theoretical analysis with these experimental studies. Based on the major features or characteristic lengths extracted from the surface structures of the abovementioned works, the structural comparison in the form of scattered points is shown in Figure S8. It can be seen that most of these surface configurations satisfy the continuous dropwise condensation criteria, which is illustrated by the central green region, proposed by us. In addition, the nanofibers in Ref 46 are ~20 μm - 30 μm tall with a high aspect ratio so that condensate droplets may be initially formed in the Cassie state instead of in the partial wetting (PW) morphology, which is not in accord with the situation of the PW-Cassie transition as discussed in our analysis.

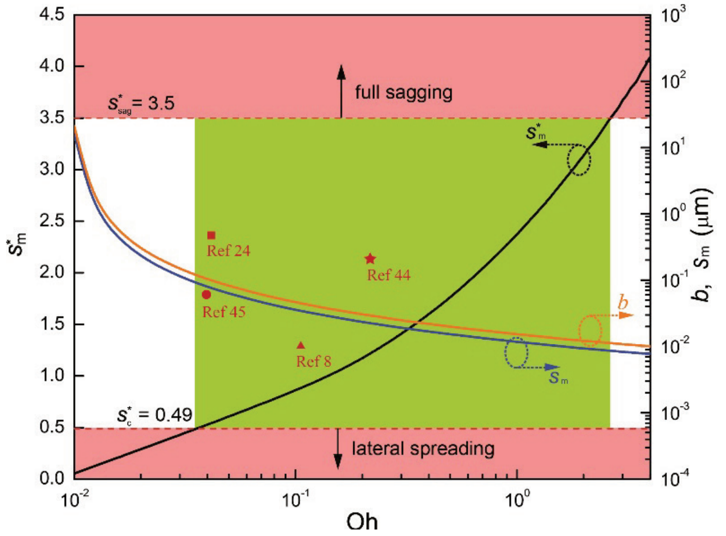

Figure S8. Validation of our resistant energy analysis by comparing with the experimental studies of references 8, 24, 44 and 45 in the manuscript.

References:

1 Blake, T. D., Clarke, A., DeConinck, J. and deRuijter, M. J. Contact angle relaxation during droplet spreading: Comparison between molecular kinetic theory and molecular dynamics. *Langmuir* **13**, 2164-2166, 1997.

2 Blake, T. D. and Haynes, J. M. Kinetics of Liquid/Liquid Displacement. *J Colloid Interf Sci* **30**, 421-423, 1969.

3 de Ruijter, M. J., Blake, T. D. and De Coninck, J. Dynamic wetting studied by molecular modeling simulations of droplet spreading. *Langmuir* **15**, 7836-7847, 1999.

4 de Ruijter, M. J., De Coninck, J., Blake, T. D., Clarke, A. and Rankin, A. Contact angle relaxation during the spreading of partially wetting drops. *Langmuir* **13**, 7293-7298, 1997.

5 Goldstein, H. *Classical Mechanics*. Pearson Education India, 1965.

NOMENCLATURE

r	m	Surface roughness / Radius
b	m	First-tier width
h	m	First-tier height / latent heat
l	m	First-tier pitch size
f		Solid fraction
e		Relative strength of line tension
G	J	Activation free energy
T	K	Temperature
W	J	Work
A	m^2	Area
F	N	Force
E	J	Energy
t	s	Time
U	m/s	Velocity
Oh		Ohnesorge number
P	N/m^2	Pressure
m	kg	mass
k	Hz	Molecular displacement frequency
R		Radial coordinate
k_B	m^2kg/ s^2K	Boltzman constant
N_s	$1/ m^2$	Nucleation site density

Special character

ρ	kg/m^3	Density of liquid
μ	$N\cdot s/m^2$	Dynamic viscosity
σ	N/m	Surface tension
Ω	m^3	Tail volume
θ		Young's contact angle
ϑ		Azimuthal coordinate
ζ	$kg/m\cdot s$	Contact line friction
v	m^3/kg	Specific volume
Φ	$N/m^2\cdot s$	Dissipation function
λ		Mean displacement

$\mathcal{R}$		Energy ratio
κ	1/m	Surface curvature of liquid

Subscripts

c	Cassie State / Cavity
w	Wenzel state / Wall
f	First-tier
n	Nano/second-tier
e	Equilibrium
l	Liquid
v	Vapor
adh	Adhesion
vis	Viscous
sat	Saturation
b	Base
lv	Liquid-vapor
sv	Solid-vapor
sl	Solid-liquid
d	Dynamic
0	Static
A	Advancing
